# The STOP-Bang Test Is Useful for Predicting the Severity of Obstructive Sleep Apnea

**DOI:** 10.31662/jmaj.2020-0002

**Published:** 2020-10-02

**Authors:** Hideto Oshita, Noriaki Ito, Misato Senoo, Kunihiko Funaishi, Yasuyuki Mitama, Ken Okusaki

**Affiliations:** 1 Department of Internal Medicine, Mihara Medical Association Hospital, Mihara, Japan; 2 Department of Respiratory Medicine, Hiroshima University Hospital, Hiroshima, Japan

**Keywords:** obstructive sleep apnea, sleep-disordered breathing, STOP-Bang test, apnea hypopnea index

## Abstract

**Introduction:**

The STOP-Bang test was used to detect patients at high risk of obstructive sleep apnea (OSA). We evaluated the usefulness of the STOP-Bang test for predicting the severity of OSA in Japanese patients.

**Methods:**

We retrospectively evaluated the patients who performed full polysomnography at the Mihara Medical Association Hospital. We evaluated the correlation between the STOP-Bang score and the apnea hypopnea index (AHI) using Spearman's rank correlation analysis. We then used multivariate analyses to examine the independent risk factor for severe OSA (AHI ≥ 30/hr).

**Results:**

One hundred seven patients were diagnosed as no (n = 5), mild (n = 17), moderate (n = 30), and severe (n = 55) OSA. The median age was 67 years old (range: 35-84), and 73 of the 107 patients were males. The correlation coefficient between the STOP-Bang score and AHI was 0.701 (P < 0.001). A STOP-Bang score ≥ 5 had sensitivity of 80.0% and specificity of 76.9% for detecting severe OSA. A STOP-Bang score ≥ 5 and BMI ≥ 30 kg/m^2^ were the independent risk factor for severe OSA.

**Conclusions:**

The STOP-Bang score correlates with AHI and is useful for predicting OSA severity. Polysomnography should be performed actively for the patients with high STOP-Bang scores.

## Introduction

Obstructive sleep apnea (OSA) is a common public health problem, and it has been shown to be associated with an increased incidence of hypertension ^(1)^, cardiovascular disease ^(2)^ and cerebrovascular disease ^(3)^. In addition, OSA is associated with an increased risk of traffic accidents and work-related injuries ^(4)^. Diagnosing latent OSA is clinically relevant because untreated OSA has been associated with increased mortality ^(5)^.

The STOP-Bang test, developed in 2008 by Chung F. et al., has been widely known as a sensitive, simple and easy-to-remember screening tool for OSA ^(6)^. The STOP-Bang acronym stands for: snoring history, tired during the day, observed stop of breathing while sleeping, high blood pressure, BMI > 35 kg/m^2^ (or 30 kg/m^2^), age > 50 years, neck circumference > 40 cm and male gender (Table 1). The STOP-Bang test was originally established to screen for OSA in a Canadian surgical population and has been used and validated in a preoperative population ^(7)^, sleep clinic population ^(8), (9)^, bus-driver population ^(10)^ and general population ^(11)^ to detect patients at high risk of OSA. Recently, we reported that the Japanese translated version of the STOP-Bang test was useful for risk assessment of OSA in Japanese inpatients ^(12)^.

**Table 1.** The STOP-Bang Test.

	Yes	No
STOP		
Do you snore loudly (louder than talking or loud enough to be heard through closed doors)?	1	0
Do you often feel tired, fatigued, or sleepy during daytime?	1	0
Has anyone observed you stop breathing during your sleep?	1	0
Do you have or are you being treated for high blood pressure?	1	0
BANG		
BMI > 30 kg/m^2^ (or 35 kg/m^2^)?	1	0
Age over 50 years old?	1	0
Neck circumference > 40 cm?	1	0
Gender: male?	1	0

Total of scores (out of 8)

Although there are several reports on the relationship between the STOP-Bang test and OSA severity, such a relationship has not been as well validated as the usefulness of this test in OSA screening. The purpose of this study was to validate the correlation between the STOP-Bang score and AHI and the detectability of the severe OSA in Japanese.

## Materials and Methods

### Study design and patients

The population of this retrospective study consisted of consecutive subjects who underwent full polysomnography (PSG) for diagnosis of OSA between April 2017 and June 2019 at the Department of Internal Medicine, the Mihara Medical Association Hospital. The clinical data were collected from electronic medical records. The ethics committee of the Mihara Medical Association Hospital approved this study (no. 291201), and written informed consent was obtained from the patients.

### The questionnaire

The questionnaire including demographic information (age and gender), symptoms (snoring, apnea, and daytime drowsiness) and comorbidities (hypertension, diabetes mellitus, hyperlipidemia, cardiovascular disease, and cerebrovascular disease) was filled by all subjects. Anthropometric parameters (body weight, height, and neck circumference) were measured by a nurse or doctor. The Epworth sleepiness scale (ESS) is an eight-item questionnaire used to measure daytime sleepiness. The questionnaire has a four-point Likert response format, and the score ranges from 0 to 24. ESS score ≥ 11 indicates excessive daytime sleepiness and high risk of OSA ^(13)^. Contents of the Japanese translated version of the STOP-Bang test ^(14)^ and ESS were mixed into the questionnaire, and STOP-Bang score and ESS were calculated after the diagnosis process was completed. We used 30 kg/m^2^ as the cutoff value for BMI according to previous studies in the Asian population ^(9), (12), (15)^.

### Polysomnography

PSG (PSG-1100, Nihon Kohden) included recordings of six electroencephalogram channels: bilateral electro-oculograms, chin and tibialis electromyogram, electrocardiogram, airflow by nasal pressure transducer and oronasal thermocouples, chest and abdominal wall motion by piezo electrodes, and oxygen saturation by pulse oximeter. Respiratory events were manually scored according to the American Association of Sleep Medicine guideline 2015 ^(16)^ by the clinical laboratory technician using the dedicated software. AHI was defined as the total number of apneas and hypopneas per hour of sleep. The severity of OSA based upon the AHI was categorized into four groups: none (AHI < 5/h), mild (5 ≤ AHI < 15/h), moderate (15 ≤ AHI < 30/h), and severe (AHI ≥ 30/h).

### Data analysis

The patients were divided by presence or absence of severe OSA, and clinical features obtained from the questionnaire were compared. We present continuous variables as median and range and categorical variables as numbers and percentages. We compared continuous variables by Wilcoxon rank-sum test. We compared categorical variables using the χ^2^ test when appropriate; otherwise, we used Fisher’s exact test.

The receiver operating characteristic (ROC) curve analysis was performed to evaluate the diagnostic value of the STOP-Bang test for detecting severe OSA and determining the best cutoff value. We used Spearman's rank method to examine the correlation between the STOP-Bang score or ESS and AHI. To detect the independent risk factor of severe OSA, we performed multivariate logistic regression analyses with backward elimination using the categorical variables that had shown statistical significance in univariate analyses.

All analyses were performed using EZR (Saitama Medical Center, Jichi Medical University, Saitama, Japan) ^(17)^, a graphical user interface for R statistical programming software (The R Foundation, Vienna, Austria). We regarded a P value under 0.05 as statistically significant.

## Results

One hundred nine subjects underwent full PSG as diagnosis for OSA during the inclusion period. Two subjects were excluded because of lack of clinical data (n = 1) and diagnosis of central sleep apnea (n = 1). Therefore, 107 patients were finally included in our study and categorized into 4 groups: no (n = 5), mild (n = 17), moderate (n = 30), and severe (n = 55) OSA on the basis of AHI.

In the study population, the median age of 67 years (range 35-84 years), and 68.2% of the patients were males, with median BMI of 26.4 kg/m^2^ (range: 17.3-42.3 kg/m^2^), and a with median neck circumference of 38.5 cm (range: 31-47 cm). Descriptive characteristics of the study and comparison between the patients with and without severe OSA are displayed in Table 2. The patients with severe OSA had a higher BMI and thicker neck circumference than the patients without severe OSA. They also had a higher prevalence of hypertension, hyperlipidemia and OSA symptoms such as snoring and apnea. The STOP-Bang score was significantly higher in the patients with severe OSA.

**Table 2.** Characteristics of the Study Population and Comparison between the Patients with Mild-to-moderate OSA and Those with Severe OSA.

	Total	Without severe OSA	With severe OSA	P value
n	107	52	55	
**Continuous variables, median [range]**				
Age, years old	67 [35–84]	64 [35–81]	69 [35–84]	0.02
Height, cm	164.0 [141.0–181.2]	164.0 [141.0–181.2]	164.0 [142.0–180.0]	0.87
Body weight, kg	69.0 [42.0–118.0]	64.6 [42.0–103.0]	73.2 [45.0–118.0]	0.002
BMI, kg/m^2^	26.4 [17.3–42.3]	23.9 [17.3–40.6]	28.3 [17.7–42.3]	< 0.001
Neck circumference, cm	38.5 [31.0–47.0]	38.0 [31.0–43.0]	40.0 [32.0–47.0]	< 0.001
Apnea hypopnea index, /hr	31.0 [1.9–75.9]	16.5 [1.9–27.9]	48.6 [30.5–75.9]	< 0.001
Epworth sleepiness scale	6 [0–22]	6 [0–22]	7 [0–21]	0.17
STOP-Bang score	5 [2–8]	3.5 [2–7]	5 [2–8]	< 0.001
**Categorical variables, n (%)**				
Male	73 (68.2)	33 (63.5)	40 (72.7)	0.41
Age ≧ 50 years old	97 (90.7)	45 (86.5)	52 (94.5)	0.19
BMI≧30 kg/m^2^	34 (31.8)	8 (15.4)	26 (47.3)	< 0.001
Neck circumference ≧ 40cm	39 (36.4)	11 (21.2)	28 (50.9)	0.002
Tired during the day	61 (57.5)	26 (50.0)	35 (64.8)	0.17
Snoring history (%)	70 (65.4)	27 (51.9)	43 (78.2)	0.005
Observed apnea while asleep	40 (37.7)	13 (25.0)	27 (50.0)	0.01
Hypertension	71 (66.4)	29 (55.8)	42 (76.4)	0.03
Hyperlipidemia	50 (46.7)	18 (34.6)	32 (58.2)	0.02
Diabetes mellitus	39 (36.4)	19 (36.5)	20 (36.4)	1
Cardiovascular disease	25 (23.4)	13 (25.0)	12 (21.8)	0.82
Cerebrovascular disease	10 (9.3)	5 (9.6)	5 (9.1)	1
STOP-Bang score ≧ 5	56 (52.3)	12 (23.1)	44 (80.0)	< 0.001

BMI, body mass index; OSA, obstructive sleep apnea

**Figure 1.**
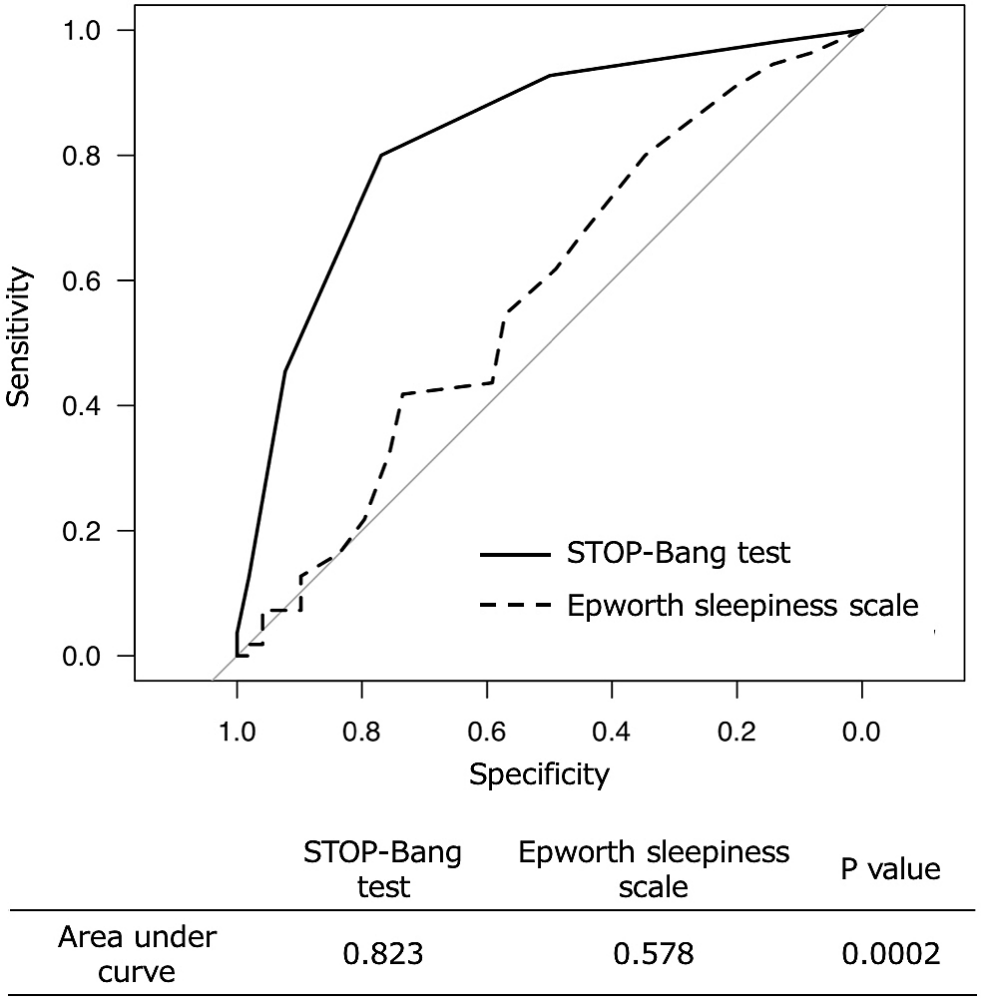
The receiver operating characteristic curve analysis of the STOP-Bang test and Epworth sleepiness scale for detecting severe OSA.

In Figure 1, we presented the ROC curves of the STOP-Bang test and ESS for detecting severe OSA. The STOP-Bang test showed statistically significantly higher area under curve (AUC) than ESS. We determined the best cutoff value to be five points of the STOP-Bang score for detecting severe OSA and resulted in sensitivity of 80% and specificity of 76.9%.

**Figure 2.**
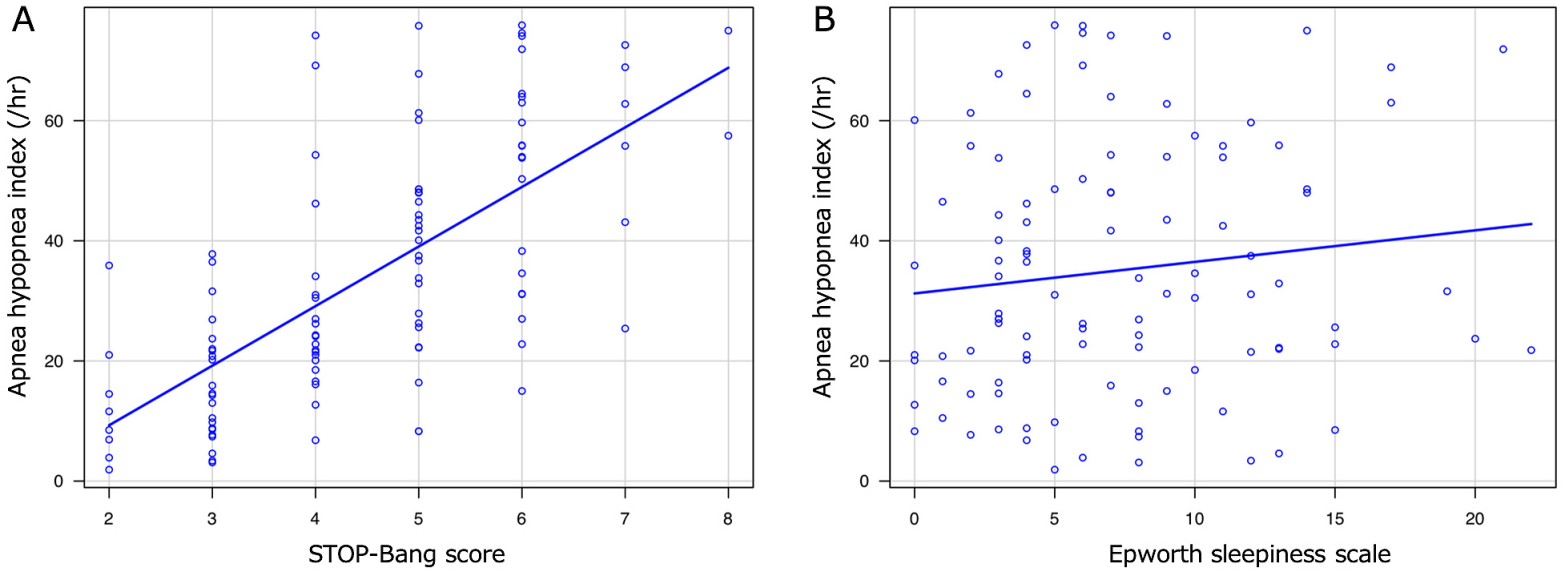
The correlation between the apnea hypopnea index (AHI) and the STOP-Bang test (A) or Epworth sleepiness scale (B). The correlation coefficients between the STOP-Bang score or ESS and AHI were 0.701 (P < 0.001) and 0.137 (P = 0.17), respectively.

The AHI of the patients according to their STOP-Bang score and ESS are presented in Figure 2. The Spearman's rank sum test showed significantly correlation between the STOP-Bang score and AHI (correlation coefficient: 0.701, P < 0.001).

Finally, we performed multivariate logistic regression analyses with backward elimination using a STOP-Bang score ≥ 5, BMI ≥ 30 kg/m^2^, hypertension, hyperlipidemia, observed apnea while sleeping, neck circumference ≥ 40 cm, and snoring history and found that the STOP-Bang score ≥ 5 and BMI ≥ 30 kg/m^2^ was an independent risk factor of severe OSA (Table 3).

**Table 3.** The Multivariate Logistic Regression Analyses for Detecting Severe OSA.

	Odds ratio (95% confidence interval)	P value
STOP-Bang score ≧ 5	11.90 (3.10–45.4)	0.0003
BMI ≧ 30 kg/m^2^	3.25 (1.03–10.30)	0.045
Hyperlipidemia	2.12 (0.78–5.75)	0.14
Observed apnea while asleep	2.14 (0.72–6.34)	0.17
Neck circumference ≧ 40 cm	0.62 (0.15–2.46)	0.49

BMI, body mass index; OSA, obstructive sleep apnea

## Discussion

Although OSA is a very common disease, only a few cases are diagnosed and receive treatment. Marin et al. reported that severe-OSA patients have higher incidence of fatal and non-fatal cardiovascular events than mild-moderate-OSA patients ^(18)^. Especially, untreated severe OSA patients should get a poor prognosis and be diagnosed and treated as early as possible.

Although the STOP-Bang test is a useful questionnaire for risk assessment of OSA, low specificity and low positive predictive value have been pointed out ^(19)^. Because many normal subjects and mild OSA patients also fall under the STOP-Bang ≥ 3, performing full PSG for all subjects with STOP-Bang ≥ 3 might be a waste of effort and cost. Several researchers, therefore, focused on the usefulness of the STOP-Bang test in predicting the severity, not in predicting the presence of OSA. Farney et al. and Chung et al. demonstrated that as the STOP-Bang score increased, the probability of having more severe OSA also increased ^(20), (21)^. However, the relationship between the STOP-Bang score and the severity of OSA is not as well established as the usefulness in OSA screening.

In the group of Japanese patients who underwent PSG, our result revealed that the STOP-Bang score correlated well with AHI and suggested that the STOP-Bang test was useful for predicting OSA severity. Notably, the STOP-Bang test was overwhelmingly better than ESS, which is widely used as a sleepiness index, in correlation with AHI and in the diagnostic value of detecting severe OSA. In particular, the STOP-Bang score > 5 showed sufficient diagnostic value in the detection of severe OSA. A high STOP-Bang score could have the clinical significance that PSG should be performed preferentially.

We recognize that this study has several limitations. First, it is a single-center study, and the number of cases might not be sufficient to generalize the results. Validation in another large cohort is required. Second, this study evaluated only patients who underwent PSG on their own will because of suspected OSA based on symptoms or physical features. This causes the selection bias, and the results cannot be easily generalized to subjects who are not concerned about their sleep.

Despite several limitations, our results suggest that the STOP-Bang test can estimate the OSA severity, and PSG should be strongly recommended for a patient with a high STOP-Bang score. The STOP-Bang test is a tool that many clinicians should know to find poor prognostic OSA patients early.

## Article Information

### Conflicts of Interest

None

### Author Contributions

Hideto Oshita conceptualized and designed the study, drafted the initial manuscript, and approved the final manuscript as submitted.

Noriaki Ito, Misato Senoo, and Kunihiko Funaishi coordinated the data collection and approved the final manuscript as submitted.

Yasuyuki Mitama and Ken Okusaki supervised this study, critically reviewed the manuscript, and approved the final manuscript as submitted.

### Approval by Institutional Review Board (IRB)

The study was approved by the Ethics Committee of the Mihara Medical Association Hospital (approval code: 291201).
